# Supplementary material for: A New Staining Method Using Methionyl-tRNA Synthetase 1 Antibody for Endoscopic Ultrasound-Guided Fine-Needle Aspiration Cytology of Pancreatic Cancer
Source: Diagnostics (Basel). 2025 Jul 15;15(14):1783. doi: 10.3390/diagnostics15141783 (PMC12293401; doi:10.3390/diagnostics15141783)

Supplementary Materials

This Supplementary Materials has been provided by the authors to give readers additional information regarding their work.

Supplement to: A New Staining Method Using Methionyl-tRNA Synthetase 1 Antibody for Endoscopic Ultrasound-Guided Fine-Needle Aspiration Cytology of Pancreatic Cancer

Sung Ill Jang, See Young Lee, Ji Hae Nahm, Jae Hee Cho, Jung Hyun Jo, Chan Min Jung, Beom Jin Lim, Jin Hong Lim, Hyung Sun Kim, Su Yun Lee, In Young Hong, Sunghoon Kim, Dong Ki Lee

Contents

1. Supplementary Materials and Methods

1.1 Cell cultures: page 1

2. Supplementary Results: page

Supplementary Figure S1: page 3

Supplementary Figure S2: page 4

1.1 Cell cultures

PANC-1 pancreatic cancer and CT 26 colon cancer cell lines were obtained from Leibniz-Institut DSMZ (Braunschweig, Germany) and American Type Culture Collection (Manassas, VA, USA) and used as positive and negative controls during MARS1 IF staining, respectively. PANC-1 cells were cultured in RPMI-1640 (Gibco BRL) with 10% fetal bovine serum (Hyclone; Thermo Fisher Scientific), and CT 26 cells were cultured in Dulbecco's modified Eagle medium (Hyclone) with 50 μg/mL penicillin/streptomycin (Hyclone). Both lines were grown in 5% CO_2 at 37°C. To obtain liquid-based cytology slides for MARS1 IF staining, confluent cells were treated with 0.05% trypsin-EDTA (Hyclone) and prepared using ThinPrep (Cytyc Co.) according to manufacturer instructions.

Supplementary Figure S1. Representative positive immunofluorescence (IF)-staining pattern of methionyl-tRNA synthetase 1 (MARS1). **A-C,** Results for the PANC-1 cell line (positive control) demonstrating a cytoplasmic IF-staining pattern. Three representative pancreatic cancer cell samples are shown, demonstrating intense cytoplasmic MARS1 IF staining. DAPI represents nuclear staining with 4',6-diamidino-2-phenylindole, MARS1 represents MARS1 IF staining, and Merge represents the combined MARS1 and DAPI staining images.

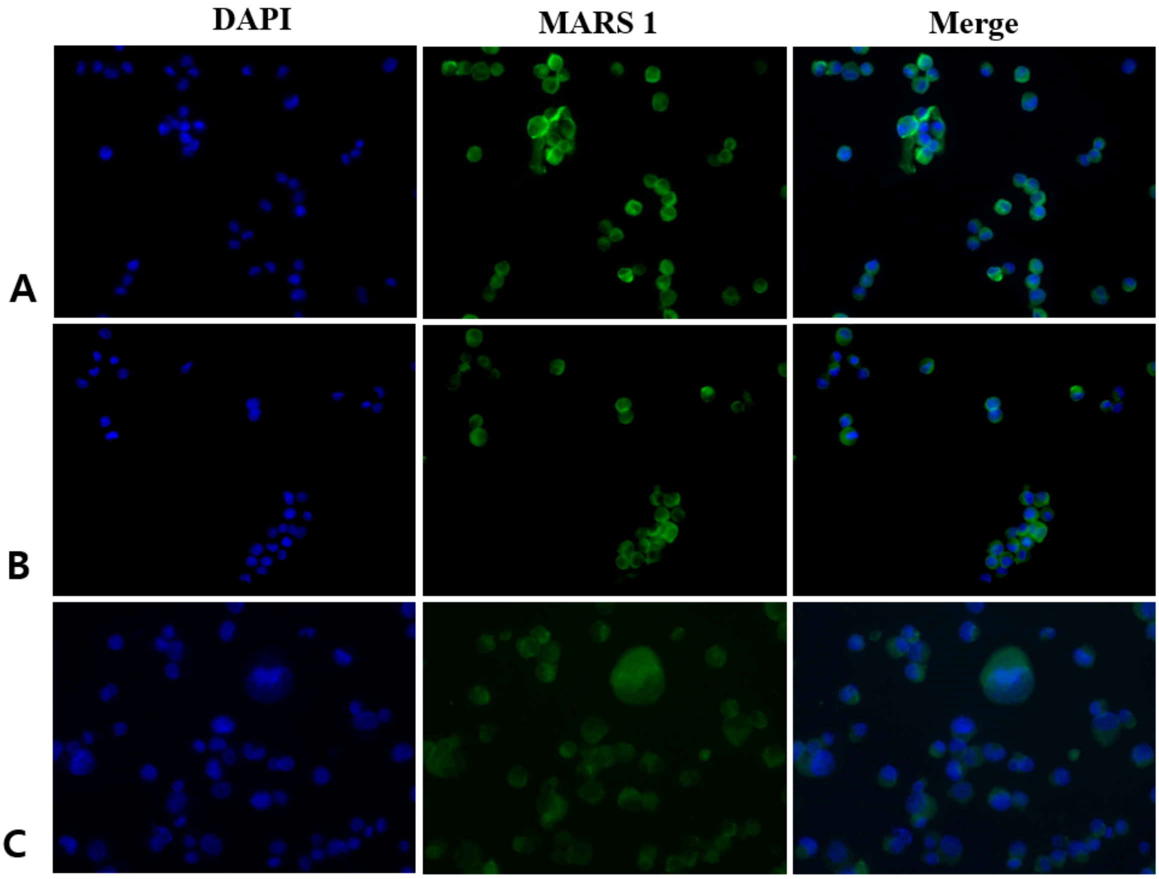

Supplementary Figure S2. Representative negative immunofluorescence (IF)-staining patterns for methionyl-tRNA synthetase 1 (MARS1). **A-C,** Results for the colon cancer (CT 26) cell line (negative control) demonstrating weak or dim fluorescent cytoplasmic signal in epithelial cells (considered negative). DAPI represents nuclear staining with 4',6-diamidino-2-phenylindole, MARS1 represents MARS1 IF staining, and Merge represents the combined MARS1 and DAPI staining images.

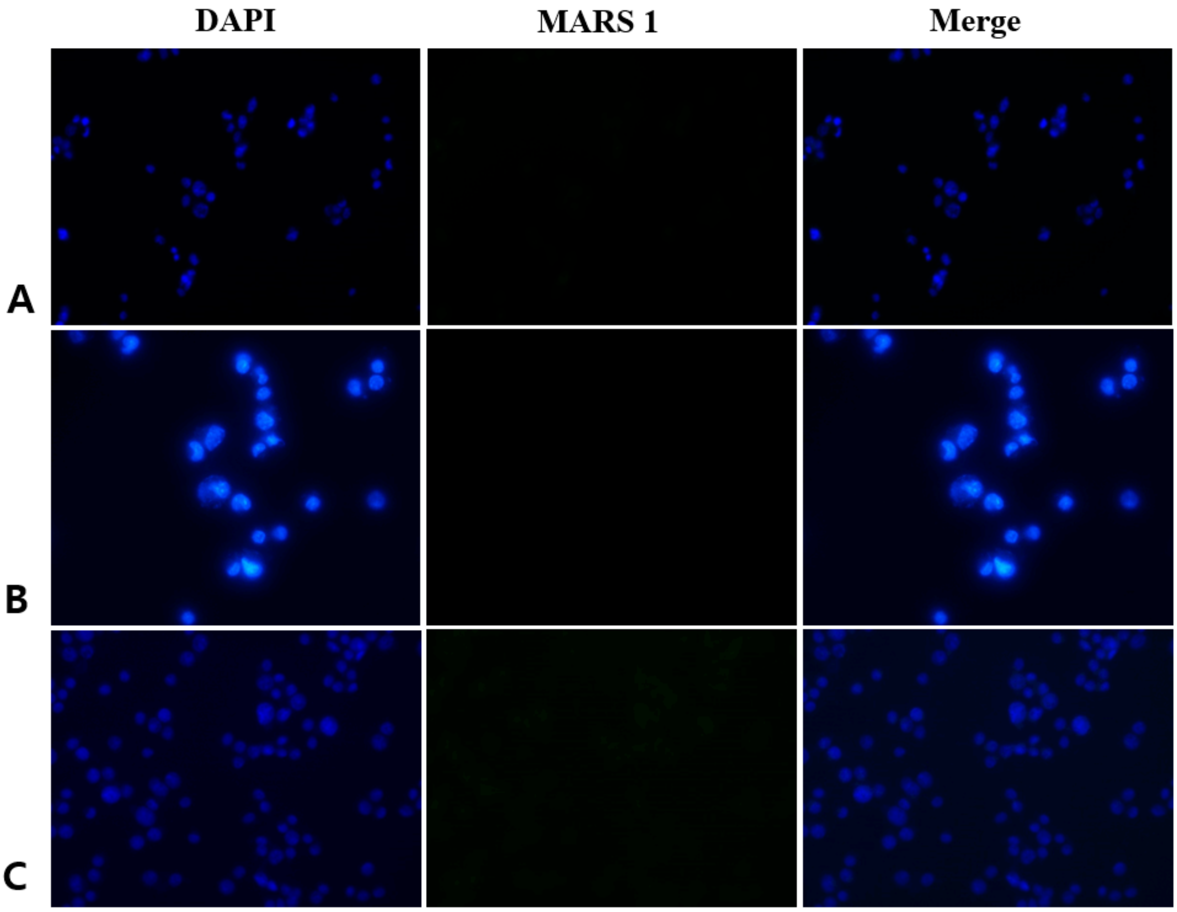

Supplement: Supplementary file 1 [file diagnostics-15-01783-s001.zip › diagnostics-3678144-Supplementary.pdf]
